# Chromosome 18q deletion and Smad4 protein inactivation correlate with liver metastasis: a study matched for T- and N- classification

**DOI:** 10.1038/sj.bjc.6603460

**Published:** 2006-11-07

**Authors:** T Tanaka, T Watanabe, Y Kazama, J Tanaka, T Kanazawa, S Kazama, H Nagawa

**Affiliations:** 1 Department of Surgical Oncology, University of Tokyo, 7-3-1 Hongo, Bunkyo-Ku, Tokyo, Japan; 2 The Department of Surgery, Graduate School of Medicine, Teikyo University, 2-11-1 Kaga, Itabashi-Ku, Tokyo, Japan

**Keywords:** colorectal cancer, Smad4, 18q, LOH, liver metastasis

## Abstract

Smad4 protein, whose gene is coded at chromosome 18q21.1, is an important tumour suppressor that mediates transforming growth factor-beta. It has been reported that inactivation of the *Smad4* gene and allelic loss of chromosome 18q correlate with liver metastasis and poorer prognosis in colorectal cancers. Utilising a recently developed method of immunohistochemical staining for Smad4 protein, we focused on the specific impact of Smad4 protein expression on liver metastasis in colorectal cancer. We also evaluated the association between chromosome18q deletion and liver metastasis. We selected 20 colorectal cancers with liver metastasis for the experimental group, and 20 cases without liver metastasis for the control. In order to exclude the influence of lymph node metastasis, all cases were lymph node negative. In addition, the two groups were matched for tumour depth, tumour differentiation and tumour location. We compared the expression level of Smad4 protein immunohistochemically in these 20 matched pairs. We also compared the loss of heterozygosity status at chromosome 18q in these 20 matched pairs. Immunohistochemical staining revealed a significant difference (*P*=0.024) in the level of Smad4 protein between the two groups. We also observed a significantly different (*P*=0.0054) ratio of allelic deletion at chromosome 18q21. Smad4 protein expression level and allelic loss at 18q21 are associated with the process of liver metastasis in colorectal cancers evaluated when excluding clinical and pathological features except for liver metastasis.

Colorectal cancer develops through multistep genetic alterations, involving several oncogene and tumour suppressor genes (Vogelstein *et al*, 1988, 1989; Benhattar *et al*, 1993; Kinzler and Vogelstein, 1996; Span *et al*, 1996; Kambara *et al*, 2004; Nassif *et al*, 2004). Loss of heterozygosity (LOH) is one of the major types of genetic inactivation, and the long arm of chromosome 18 is the most frequently deleted region in colorectal cancers. To date, many reports suggest that this deletion is a molecular predictor that affects survival (Fearon *et al*, 1990; Jen *et al*, 1994; Chung, 1998; Lanza *et al*, 1998; Ogunbiyi *et al*, 1998; Jernvall *et al*, 1999; McLeod and Murray, 1999; Sarli *et al*, 2004). We too reported that the allelic deletion of chromosome 18q was associated with poorer prognosis in stage III colon cancer after adjuvant chemotherapy (Watanabe *et al*, 2001, 2006). These reports suggest that there might be tumour suppressor genes located at chromosome 18q, which has a strong influence on survival. *Smad4* gene, which mediates the intracellular signalling pathway of transforming growth factor (TGF)-beta receptor, has been detected as one of the target genes at 18q21 (Wang *et al*, 1995; Eppert *et al*, 1996; Hahn *et al*, 1996; Thiagalingam *et al*, 1996; Carethers *et al*, 1998; Markowitz, 2000; Fink *et al*, 2003; Alazzouzi *et al*, 2005; Li *et al*, 2005). Recently, an immunohistochemical method for evaluating Smad4 protein has been developed and several studies found higher frequency of Smad4 protein inactivation in the cases with liver metastasis and in the cases presenting unfavourable survival (Maitra *et al*, 2000; Alazzouzi *et al*, 2005).

Currently, recurrences of colorectal cancers appear mainly as lymph node metastasis or liver metastasis. This means that both lymph node metastasis and liver metastasis have an influence on survival after surgery. Furthermore, tumour depth and tumour differentiation cannot be ignored when thinking of the malignancy potential of the tumour. However, in all the previous reports discussing the correlation of Smad4 protein and 18q deletion with survival, tumour depth, lymph node metastasis and tumour differentiations were not matched. Under such conditions, it was impossible to detect which of these clinical and pathological features that determine survival were truly associated with Smad4 protein expression and 18q deletion.

In this report, we attempted to focus on the specific influence of Smad4 protein expression and 18q deletion on the development of liver metastasis. In order to achieve this aim, we selected only lymph node negative cases so as to exclude the influence of lymph node invasion. Furthermore, we match tumour depth, tumour differentiation and tumour locations, which have been reported to influence LOH status and microsatellite instability. To the best of our knowledge, this is the first report to assess the specific impact of Smad4 protein and 18q deletion on the development of liver metastasis under these conditions, which enabled us to exclude the influence of clinical and pathological features other than liver metastasis.

## MATERIALS AND METHODS

### Patient samples

Specimens were obtained during routine operations at the Department of Surgical Oncology, Tokyo University Hospital, from January 1980 to July 2005. Cases of familial adenomatous polyposis and hereditary nonpolyposis colorectal carcinoma were excluded from this study. No patient received cytotoxic therapy before surgery. The study was approved by the Ethics Committee of Tokyo University Hospital and patients gave their written informed consent for the use of the specimens in advance.

There were 2783 cases of colorectal cancers operated on in our hospital during this period and 261 cases were positive for liver metastasis at the time of surgery. We selected 20 cases out of them, which are confirmed negative for lymph node metastasis.

First, these 20 cases were assigned to the *liver-metastasis* (+) group. Next, 20 cases negative for liver metastasis and negative for lymph node metastasis were assigned to the *liver-metastasis* (−) group. The pathological T-classification, tumour location and tumour differentiation of both groups were matched.

The clinical and pathological features of both groups are shown in Table 1. Of the 40 patients in the two groups, 29 were male and 11 were female. The age distribution was from 26 to 79 years. There was no statistically significant difference in the distribution of sex and age between the *liver-metastasis* (+) and *liver-metastasis* (−) groups. Pathological staging in each case was according to the UICC/TNM classification. T-classification and tumour differentiation in each group were the same, as follows: T1, one (5%); T2, one (5%); T3, 17 (85%); T4, one (5%); well-differentiated adenocarcinoma, 19 (95%); poorly differentiated adenocarcinoma, one (5%). The distribution of tumour location was also matched in both groups: right colon, two (10%); left colon, four (20%); rectum, 14 (70%). The mean and median follow-up period were 1438 days and 1014 days for *liver-metastasis* (−), during which no case of *liver-metastasis* (−) has been confirmed not to experience recurrence.

### Immunohistochemical staining

All the samples for immunohistochemical analysis were obtained from paraffin-embedded specimens. Serial sections were cut at a thickness of 3 *μ*m for both Smad4 immunostaining and haematoxylin–eosin (H&E) staining.

The sections were deparaffinized with xylene and dehydrated with 98% ethanol, placed in 0.01 M sodium citrate buffer (pH 6.0), and heated in a microwave oven for three 7-min cycles (500 W). After washing three times in PBS, endogeneous peroxidase activity was inhibited by incubation with 0.3% hydrogen peroxide in methanol for 20 min. Biotinylated rabbit anti-mouse immunoglobulin and SAB complex which were supplied commercially (Histofine SAB-PO(M) kit, Nichirei, Tokyo, Japan) were used as reagents for the next step. The sections were incubated overnight at a temperature of −4°C with anti-SMAD4 monoclonal antibody (Smad4(B-8):sc-7966, Santa Cruz Biotechnology, Inc., CA, USA) at a dilution of 1 : 1000. Colour was then developed with diaminobenzidine solution. Sections were then lightly counterstained with a cocktail of Mayer's/Lillie-Mayer's haematoxyline and mounted.

### Evaluation of Smad4 immunostaining

All the specimen immunostained for Smad4 protein were evaluated without knowing their clinicopathological features. Smad4 staining was predominantly observed in cytoplasm.

All the normal mucosa showed immunohistochemical staining against Smad4 protein. The samples were evaluated as reported previously (Alazzouzi *et al*, 2005). To evaluate the intensity of Smad4 immunohistochemical staining, we used a semiquantitative scale, where 0=no Smad4 staining and 4 was the highest staining. The samples that stained as strong as normal mucosa were scored as 4, whereas the samples with no detectable immunohistochemical staining were scored as zero. The grades of the remaining weakly and partially stained samples were classified as previously reported (Iacobuzio-Donahue *et al*, 2000). The samples in which all the tumour cells showed positive but weaker stain than the normal mucosa were scored as 3. And the samples that showed diffuse stain were scored as 2, whereas the samples that showed focal stain were scored as 1.

**Figure 1:**
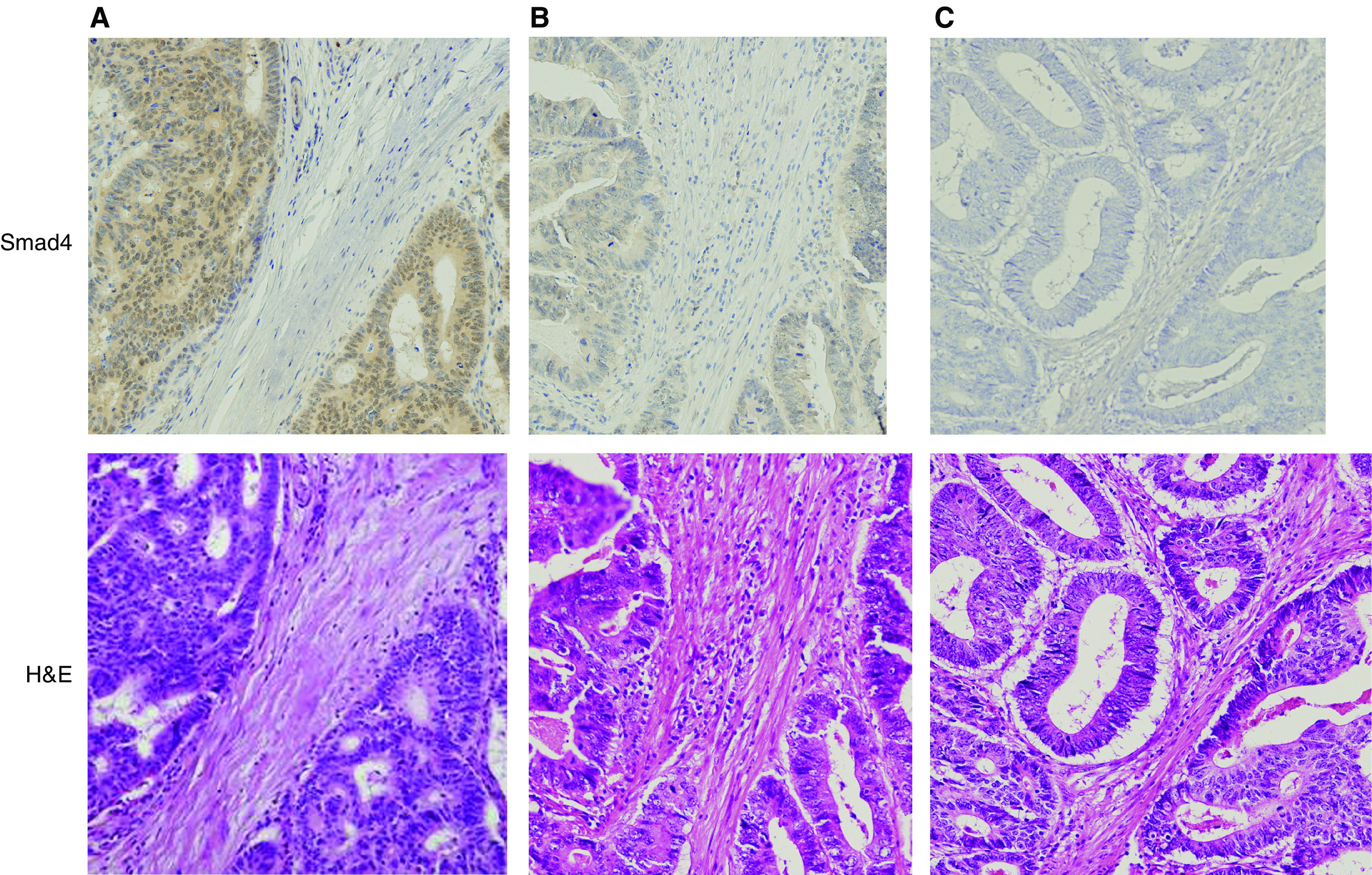
SMAD4 immunohistochemical staining of colorectal samples. (**A**) high Smad4, (**B**) low Smad4, (**C**) no Smad4. Samd4: Smad4 immunohistochemical staining. HE: corresponding samples of H&E staining.

For statistical analysis, we classified these scores into three groups: (1) no Smad4 (scored 0), (2) low Smad4 (scored 1 to 3) and (3) high Smad4 (scored 4). Representative samples of Smad4 staining are shown in Figure 1.

### Analysing the status of LOH

From the *liver-metastasis* (+) and *liver-metastasis* (−) groups, 12 T-matched pairs of tumours were available from their primary frozen specimens. These specimens were used for LOH analysis. The samples were snap-frozen in liquid nitrogen immediately after being resected during routine operations and stored at −80°C until the DNA was extracted from each specimen using DNAeasy kit (Qiagen, Tokyo, Japan). From other eight T-matched pair of samples whose frozen specimens were not stored, we also obtained DNA from paraffine-embedded specimens by microdissection techniques as we described in the previous report (Kazama *et al*, 2006), resulting in obtaining all the 20 T-matched pairs of samples in this LOH analysis.

**Figure 2:**
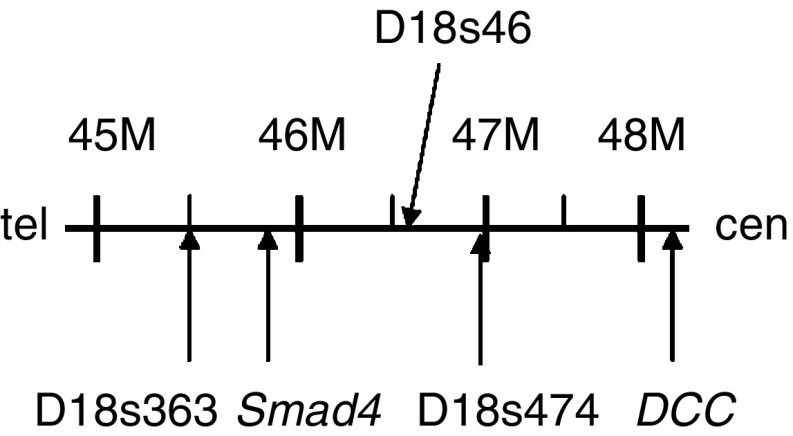
Schematic representation of the microsatellite markers analysed at 18q21.

To detect allelic loss, we employed three polymorphic markers; D18s363, D18s474 and D18s46, mapped closely to *Smad4* locus. The primer sequences were obtained at the Genome Database. The locations of the primers are shown in Figure 2. The sequence of each primer is as follow: D18s363: 5′-TTGGGAACTGCTCTACATTC-3′(sense), 5′-GCTTCATTCTCTCACTGGAT-3′(antisense); D18s474: 5′-TGGGGTGTTTACCAGCATC-3′(sense), 5′-TGGCTTTCAATGTCAGAAGG-3′(antisense); D18s46: 5′-GAATAGCAGGACCTATCAAAGAGC-3′(sense), 5′-CAGATTAAGTGAAAACAGCATATGTG-3′(antisense). Each primer pair was end-labelled with fluorochrome 6-caboxyl-fluorescein (FAM), 4,7,2′,4′,5′,7′-hexachloro-6-carboxylfluorescein (HEX) or NED (Applied Biosystems, Tokyo, Japan).

Polymerase chain reaction (PCR) was performed in 10 *μ*l reaction volumes containing 10 × PCR Gold Buffer (Applied Biosystems, Tokyo, Japan), 2.5 mM MgCl_2_, 200 *μ*l deoxynucleotide triphosphates mixture, 0.5 *μ*M of each primer, 20–40 ng of extracted DNA and 0.4 U of AmpliTaq Gold DNA polymerase (Applied Biosystems, Foster City, CA, USA). The DNA was amplified in a thermal cycler (Gene Amp PCR system 9700, Applied Biosystems) and PCR was performed according to the following protocol: 10 min at 95°C for polymerase activation; 40 cycles at 94°C for 30 s, 56°C for 1 min and 72°C for 1 min; then, an additional 30 min at 70°C. After denaturization at 95°C for 5 min, the PCR products were electrophoresed and analysed on an automated sequencer (ABI PRISM 3100 genetic analyser (Applied Biosystems)), and the fluorescent signals from alleles of different size were recorded and analysed using GeneScan version 3.1 and Genotyper version 2.1 software (both software packages, Applied Biosystems).

### Assessment of LOH

**Figure 3:**
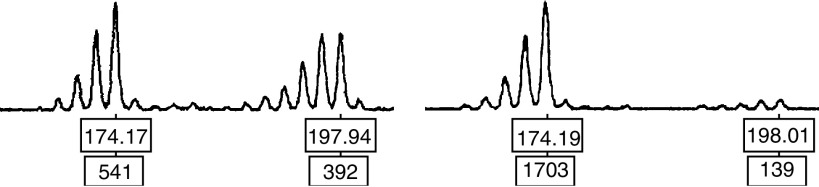
A representative example showing allelic loss. A representative example showing allelic loss: two peaks of the normal sample correspond to two alleles, and the tumour sample loses one of the peaks.

For each pair of the samples, the normal mucosal DNA was used to determine the allele size for the corresponding subject. Samples whose normal mucosa showed only a single peak were considered not informative for LOH. Samples showing two distinct peaks were considered informative and were assessed for LOH. Loss of heterozygosity was defined as a loss of one of the two peak alleles. A sample showing LOH is shown in Figure 3.

### Statistical analysis

Fisher's exact test was used to determine the significance of observed difference between the groups. A value of *P*<0.05 was considered statistically significant.

## RESULTS

### Immunochemical staining of Smad4 protein

**Figure 4:**
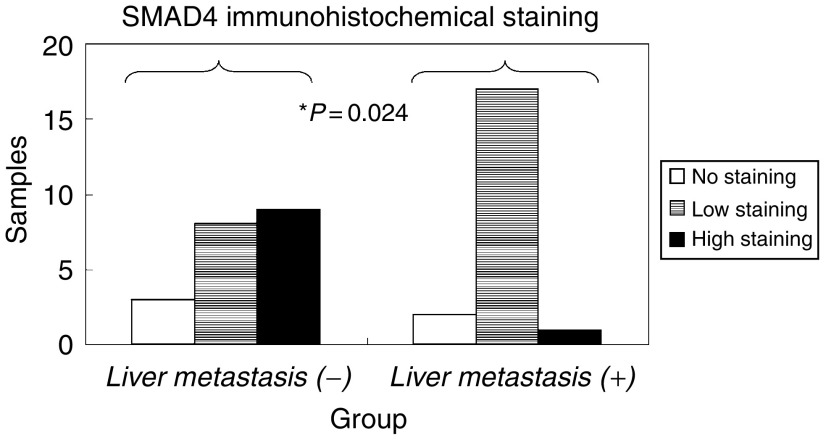
SMAD4 immunohistochemical staining of colorectal tumours. *liver-metastasis* (−): a group of tumours without liver metastasis. *liver-metastasis* (+): a group of tumours with liver metastasis.

The results of immunohistochemical staining of Smad4 protein are shown in Figure 4.

In *liver-metastasis* (−) group, nine cases (45%) were classified as high Smad4, eight (40%) as low Smad4 and three (15%) as no Smad4. In *liver-metastasis* (+) group, only one case (5%) was classified as high Smad4, while 17 cases (85%) were classified as low Smad4 and two (10%) as no Smad4. In other words, more cases showed decreased intensity for Smad4 immunohistochemical staining in *liver-metastasis* (+) group compared to *liver-metastasis* (−) group.

We statistically compared the *liver-metastasis* (+) and *liver-metastasis* (−) groups from the standpoint of Smad4 protein immunohistochemical staining and found that Fisher's exact test showed a significant difference between both the groups (*P*=0.024).

### Analysis of LOH status of Smad4

**Figure 5:**
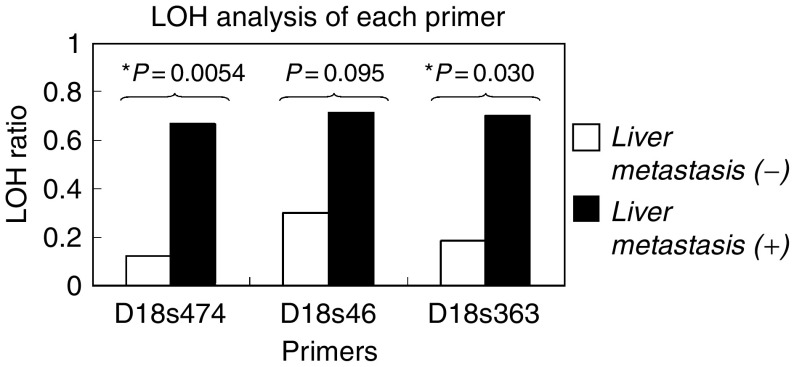
Loss of heterozygosity patterns of each primer. *liver-metastasis* (−): a group of tumours without liver metastasis. *liver-metastasis* (+): a group of tumours accompanying liver metastasis.

The results for individual primers are shown in Figure 5. The LOH ratio was defined as the number of LOH cases divided by all the informative cases in each primer. No cases in this study showed microsatellite instability.

For D18s474, 28 out of 40 cases were informative. The LOH ratios were 13% (two out of 16) in the *liver-metastasis* (−) group and 67% (eight out of 12) in the *liver-metastasis* (+) group. Fisher's exact test showed that the cases in *liver-metastasis* (+) group showed significantly higher ratio of allelic loss at D18s474 compared with those in *liver-metastasis* (−) group (*P*=0.0054). For D18s46, 24 out of 40 cases were informative, and the LOH ratios were 30% (three out of 10) in the *liver-metastasis* (−) group *vs* 71% (10 out of 15) in the *liver-metastasis* (+) group, with no significant difference (*P*=0.095) found between the groups. For the primer D18s363, 21 out of 40 cases were informative, and 18% (two out of 11) of the cases demonstrated LOH in the *liver-metastasis* (−) group *vs* 70% (seven out of 10) in the *liver-metastasis* (+) group. This result showed a statistically significant difference (*P*=0.030).

### Correlation between LOH at chromosome 18q21 and Smad4 immunohistochemical staining

**Figure 6:**
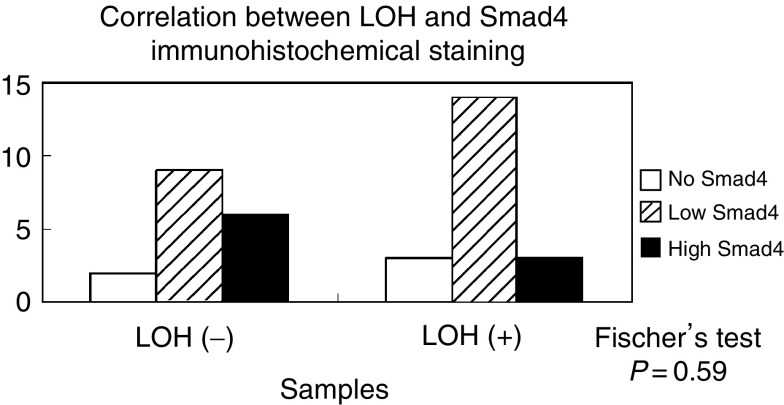
Correlation between LOH and Smad4 immunohistochemical staining.

We then examined if LOH at chromosome 18q21 had a direct effect on Smad4 protein level in 40 samples (Figure 6). Those samples that showed allelic loss in any of these three primers were classified positive for LOH. The samples in *liver-metastasis* (+) group showed more cases of low Smad4 compared to *liver-metastasis* (−) group, although statistical analysis did not show a significant difference (*P*=0.59).

## DISCUSSION

In the current study, we selected only lymph node negative cases and matched for T-classification, tumour locations and tumour differentiations between *liver-metastasis* (+)/(−) groups, and demonstrated significant statistical differences between these two groups in Smad4 protein level and chromosome 18q deletion. This is the first report to focus on the specific impact of liver metastasis on Smad4 protein expression and chromosome 18q deletion with clinical features other than liver metastasis being matched. Decreases in Smad4 protein expression and chromosome 18q deletion are known to be characteristic risk factors of liver metastasis.

It has been proven that the loss of chromosome 18q, which is present in about 70% of colorectal cancers, is related to tumour progression, recurrence and poor prognosis. Jen *et al* (1994) reported that patients with stage II colorectal cancer and chromosome 18q allelic loss show worse prognosis than those without 18q allelic loss. They pointed out that stage II colorectal cancer patients with 18q allelic loss have a similar prognosis to stage III patients. Moreover, they argued that stage II colorectal cancer patients without 18q allelic loss show similar prognosis to those with stage I patients. A study by Lanza *et al* (1998) also showed similar results in that patients with stage II disease whose tumour had no 18q allelic loss demonstrated a 5-year survival rate of 96%, while those with stage II disease and 18q allelic loss showed a 5-year survival rate of only 54%. Ogunbiyi *et al* (1998) reported that Chromosome 18q allelic loss was significantly associated with reduced disease-free and disease-specific survival in patients with stage II (*P*=0.05 and *P*=0.0156) and III disease (*P*=0.038 and *P*=0.032). We showed that microsatellite stable (MSS) patients with stage III colorectal cancer showed poor prognosis when chromosome 18q showed allelic loss (*P*=0.006) (Watanabe *et al*, 2001). We also recently argued that 18q allelic loss is a significant prognostic value in colorectal cancer (Watanabe *et al*, 2006).

In the current study, we demonstrated that there was a significant statistical association of chromosome 18q deletion with liver metastasis, which has a strong impact on survival after surgery. This study was the first to evaluate the influence on liver metastasis of 18q deletion where tumour depth, lymph node invasion, tumour differentiation and tumour location were excluded, and confirmed that 18q allelic loss is a useful marker of liver metastasis.

There have been numerous efforts to detect the target genes at chromosome 18q in colorectal carcinogenesis and cancer progression, and two putative genes have been detected. The *DCC* gene located at 18q21 was first considered to be an important target gene and to play an important role in colorectal pathogenesis (Fearon *et al*, 1990; Shibata *et al*, 1996). However, there have been several cases with 18q deletion in which no inactivation of *DCC* was observed, implying the existence of another target gene (Cho *et al*, 1994). Subsequently, *Smad4* gene was detected as another target at 18q21.1, whose mutations were detected up to 35% of colorectal cancers (Shibata *et al*, 1996).

In a previous study, the genomic inactivation of *Smad4* was reported to correlate with carcinogenesis, tumour progression and poorer prognosis in colorectal caners. Miyaki *et al* (1999) reported that they found no *Smad4* mutation in adenomas and intramucosal carcinomas, although the frequency of the mutation increased as the invasiveness of the tumour grew. Furthermore, they found a higher frequency of mutation in colorectal cancers with distant metastasis compared to those without distant metastasis. Their study strongly indicated that the *Smad4* gene correlates with malignancy in the advanced stage and is the target gene on 18q21.1, the locus indicative of tumour progression and poorer prognosis when deleted.

Recently, an immunohistochemical assay of Smad4 protein has been developed and several studies indicated that Smad4 protein inactivation affected survival. Anirban Maitra *et al* classified patients according to TNM stage and examined Smad4 protein expression for the first time. In their study, they found no Smad4 protein inactivation in stage I patients, 8% in stage II, 6% in stage III and 22% in stage IV patients, indicating that tumour progression correlates with Smad4 protein inactivation. They also classified patients into two groups according to the status of liver metastasis, and indicated a borderline significant correlation between Smad4 staining and liver metastasis (*P*=0.05) (Maitra *et al*, 2000). Alazzouzi *et al* (2005) studied 86 Dukes' C colorectal cancer patients and showed that patients with Dukes' C tumours expressing high Smad4 protein levels had significantly better overall (*P*<0.025) and disease-free (*P*<0.013) survival than patients with low levels by immunohistochemical staining. We, too, recently discussed the association between Smad4 level and prognosis in colorectal cancers. Previous reports suggested that Smad4 protein inactivation correlates with liver metastasis; however, the groups consisted of various T- and N-stage patients.

In this study, lymph node negative cases were matched for T-classification, tumour depth and tumour location and demonstrated a statistically significant difference (*P*=0.024) in Smad4 protein expression level between *liver-metastasis* (+)/(−) groups. This result confirmed more accurately the previous report that Smad4 protein inactivation is related to the process or the risk of liver metastasis.

In conclusion, our results showed that both chromosome 18q deletion and Smad4 protein expression are associated with liver metastasis in colorectal cancers, and that they both play an important role in the development of liver metastasis.

## Figures and Tables

**Table 1:** Clinicopathological features of colorectal carcinomas

**Group**	***Liver metastasis* (+)**	***Liver metastasis* (−)**	
Age	61.2±3.8	57.1±5.2	NS
*Gender*			
Male	15	14	
Female	5	6	NS
			
*T-classification*			
T1	1	1	
T2	1	1	
T3	17	17	
T4	1	1	
			
*Location*			
Right colon	2	2	
Left colon	4	4	
Rectum	14	14	
			
*Differentiation*			
Well	19	19	
Moderately	0	0	
Poorly	1	1	

*Liver metastasis* (−): a group of tumours without liver metastasis.

*Liver metastasis* (+): a group of tumours with liver metastasis.
